# Quality of life post barbed reposition pharyngoplasty in obstructive sleep apnea patients: A pre-post quasi-experimental study at secondary hospital

**DOI:** 10.5339/qmj.2025.37

**Published:** 2025-06-09

**Authors:** Frat Abbas, Mohammed Ali Kayidon Nasur, Hossam Makki, Ahmad R. AL-Qudimat

**Affiliations:** 1 ORL-Head &Neck Surgery Department, Al-Khor Hospital, Hamad Medical Corporation, Al-Khor, Qatar; 2 Surgical Research Section, Surgery Department, Hamad Medical Corporation, Doha, Qatar; 3 Department of Public Health, Health Science, Qatar University, Doha, Qatar *Email: aalqudimat@hamad.qa

**Keywords:** Functional Outcomes of Sleep Questionnaire, barbed reposition palatoplasty, quality of life

## Abstract

**Background:**

Obstructive sleep apnea syndrome (OSAS) significantly impacts patients’ quality of life (QOL) due to symptoms such as impaired sleep and reduced daily functioning. Barbed reposition pharyngoplasty (BRP) has emerged as a surgical intervention aimed at alleviating these symptoms. However, its effect on patients’ QOL remains underexplored.

**Objectives:**

To evaluate the impact of BRP surgery on a patient’s QOL with OSAS.

**Methods:**

Our study had a quasi-experimental design, which was conducted for 40 adult OSAS patients (one group) from 2015 to 2023 between men and women with inclusion criteria above 18 years old who had been diagnosed with OSAS and who had BRP surgery. We measured the impact of this surgery on patient satisfaction by correlating the subjective measures of the pre- and post-operative self-administered the Functional Outcomes of Sleep Questionnaire (FOSQ) as one of the prognostic indicators.

**Results:**

The study included 40 participants, with ages ranging from their 30s to 60s. The mean age of the participants is 44.5 years (SD ± 9.5). Mean FOSQ scores had a significant increase (*p* < 0.001) in all domains (general productivity, activity level, vigilance, social outcomes, intimate) when comparing pre-operative mean scores with post-operative mean scores. No statistically significant differences were observed in mean percentage change from baseline FOSQ when compared between males and females (*p* > 0.05) for all domains (general productivity, activity level, vigilance, social outcomes, intimate).

**Conclusion:**

BRP surgery seems to have a significant impact on patient satisfaction in OSAS patients as it reflects on their health-related QOL mood, social outcome, and daily activity and improves their quality of sleep.

## Introduction

Obstructive sleep apnea syndrome (OSAS) is a prevalent breathing disorder, characterized by either a decrease or complete cessation of airflow during sleep, affecting 4% of men and 2% of women.^1^ The prevalence of mild OSAS ranges from 3% to 28% of the adult population, while moderate to severe OSAS (defined as an apnea-hypopnea index ≥ 15) affects 1%–14%.^2^ This disorder significantly impacts health-related quality of life (QOL) by impairing normal functioning, mood, and well-being, underscoring the importance of its assessment.^3^ Untreated OSAS is associated with excessive daytime sleepiness, impaired cognitive performance, and reduced overall QOL. Additionally, it is an independent risk factor for cardiovascular morbidity and mortality.^4^

To mitigate the impact of OSAS on patients’ lives and ensure comprehensive treatment plans, various therapeutic approaches have been developed. The American Academy of Sleep Medicine recommends positive airway pressure (PAP) as the first-line treatment for all severities of OSAS. However, many patients find long-term nasal continuous PAP intolerable or are unwilling to use it,^5^ prompting them to seek surgical alternatives to alleviate their symptoms. Numerous surgical procedures have been proposed to address anatomical obstructions, particularly at the level of the oropharynx.

One such surgical technique is barbed reposition pharyngoplasty (BRP), which has recently emerged as a promising treatment for OSAS patients characterized by oropharyngeal wall collapse. This technique offers reduced morbidity and higher success rates compared to traditional uvulopalatopharyngoplasty. The use of knotless barbed sutures in oropharyngeal surgery has also shown positive results in improving the biomechanical support of soft tissues, thus preventing collapse during sleep.^6^ Based on the literature, we found several predictive factors for surgical success and evaluated various aspects of QOL in OSAS patients referred to sleep clinics.^7–9^ However, no single prognostic indicator has been identified to reliably predict improvements in health-related QOL. Recent studies have confirmed the negative impact of OSAS on patients’ QOL, emphasizing the need for more specific QOL measures for sleep disorders.^2,4,10^

One such measure is the Functional Outcomes of Sleep Questionnaire (FOSQ-10), which is widely recognized for its ability to assess the impact of sleep disorders on social, mental, and physical functioning in daily life.^11^

In this study, we aim to investigate patient satisfaction following BRP surgery for OSAS. Specifically, we will assess the impact of this surgical treatment on patients’ QOL using validated QOL questionnaires, which capture clinically relevant information and provide insight into surgical performance from a clinical perspective. We will explore patients’ perceptions of how BRP surgery has affected their QOL.

## Methods

### Design/setting

This study had a pre- and post-test design on one group of patients with snoring and sleep apnea and was carried out between 2015 and 2023. The patients were recruited from ORL-H&N surgery/snoring clinic, Hamad Medical Corporation at Al-Khor Hospital in Qatar.

### Procedure

All patients in this study underwent a comprehensive clinical evaluation for OSA, including screening polysomnography (PSG), which confirmed the diagnosis of mild to moderate OSA. Following this, each patient underwent a drug-induced sleep endoscopy to precisely identify the site of airway obstruction and evaluate the suitability of BRP as a treatment option.

To assess the impact of BRP on QOL, all participants completed the self-administered Functional Outcomes of Sleep Questionnaire (FOSQ-10) both pre-operatively and post-operatively during their outpatient visits. This questionnaire was used to evaluate changes in patients’ QOL, with a particular focus on correlating the pre- and post-operative FOSQ-10 scores as a clinical measure. This comparison allowed for the identification of prognostic indicators related to patient satisfaction and the effectiveness of the surgical intervention in improving functional outcomes.

### Sample size

The sample size was determined using a connected sample size calculator (http://www.raosoft.com) with the following criteria: a level of significance of 0.05, a confidence interval of 95%, and a response distribution of 50%. A simple random selection technique was employed to select a total of 40 patients with snoring and sleep apnea at this hospital. The number of patients invited to participate in the study was 62, with 40 of them agreeing to participate, resulting in a response rate of 64%.

### Eligibility criteria

#### Inclusion criteria

This research involved adult patients above 18 years old with snoring and sleep apnea, who visited the ORL-H&N surgery/snoring clinic in a secondary hospital seeking surgical advice for their snoring and sleep apnea problems. All those patients had a thorough comprehensive clinical sleep evaluation, were diagnosed with mild to moderate OSA syndrome, and did PSG screening pre-operatively then underwent a BRP surgery as most of them were non-compliant for pap therapy or other types of non-surgical treatment.

#### Exclusion criteria

We excluded patients with snoring and sleep apnea: (a) those under 18 years of age, (b) patients who are not diagnosed with snoring and sleep apnea, and (c) those who do not agree to participate.

### Data collection procedure and instruments

Those patients had a pre- and post-operative self-administered the Functional Outcomes of Sleep Questionnaire (FOSQ)-10 filled out by themselves in post-operative visits or over the phone. Functional Outcomes of Sleep Questionnaire in its shortest version, known as FOSQ-10, was developed in 2009 by Weaver. It was developed to evaluate the functional impact of sleepiness in daily life activities.^7^ Pre- and post-operative FOSQ-10 scores as a subjective measure to identify the prognostic indicators of patients’ satisfaction with the impact of surgery on their QOL and clinical improvement. All demographic data and the FOSQ categories will be collected. The FOSQ-10 contains 5 domains subdivided into: general productivity, 2 questions; the activity level, 3 questions; vigilance, 3 questions; social outcomes, 1 question; intimate, 1 question; and has a score from 0 to 4, with score 1 having extreme difficulty while 4 no difficulty.

### Statistical analysis

The statistical analysis was performed using SPSS (V25). Descriptive statistics were generated, including means and standard deviations for continuous variables, and percentages for categorical variables. The average correct answers for pre- and post-test items were compared using paired t-tests. The Wilcoxon signed-rank test, a non-parametric test, was employed to compare pre- and post-operative scores because the data did not meet the assumptions of normality required for parametric tests, such as the paired t-test. This test is suitable for analyzing paired data where the distribution of differences is non-normal, providing robust results for small sample sizes. The statistical significance of the connection has been established to be *p* < 0.05. However, more stringent levels (*p* < 0.001) were employed where necessary for enhanced precision.

### Ethical approval

The study obtained ethical approval after being reviewed by the Institutional Review Board (IRB) from the Medical Research Center at Hamad Medical Corporation, Doha, Qatar. Approval No. (IRB-HMC-2021-011).

## Result

### Participant characteristics

**Figure 1:**
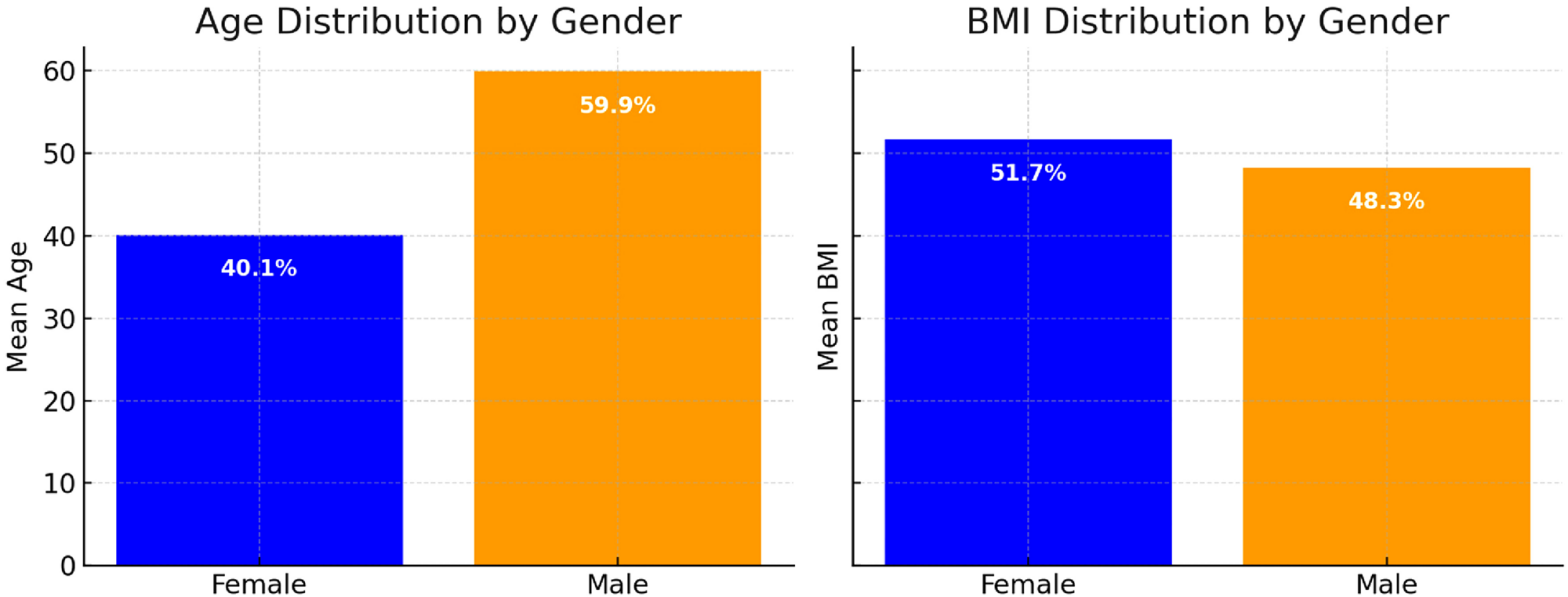
Participant characteristics.

The study included 40 participants, with ages ranging from their 30s to 60s. The mean age of the participants is 44.5 years (SD ± 9.5). The sample is composed predominantly of male participants, with a male-to-female ratio of approximately 9:1. Participants’ weights range from 74 to 102 kg; Male participants have a higher average weight than female participants, and their BMI values range from 27 to 37, Male participants have a higher average weight than female participants; indicating a range from overweight to obese categories (Figure 1).

### Correlation

Pearson correlation analysis was performed to examine and assess correlation in mean percentage change from baseline FOSQ scores for all domains (general productivity, activity level, vigilance, social outcomes, intimate) with age, and pre- and post-operative BMI values. It was found that age and post-operative BMI were inversely (however the values of the correlation coefficient were low, Pearson (r) <−0.35) correlated with general productivity, activity level, and social outcomes (*p* > 0.05). Moreover, the correlation between general productivity and activity level, vigilance, social outcomes, and intimate were found to be statistically significant and had moderate to good correlation values (Pearson (r) range 0.52–0.76, *p* < 0.001) (Table 1).

### Different means of pre- and post-operative FOSQ

The mean score in the general productivity domain at the post-operative FOSQ assessment was 7.08, compared to a pre-operative score of 5.63. Similarly, in the activity-level domain, the mean post-operative score was 10.58, compared to a pre-operative score of 8.65. In the vigilance domain, the mean post-operative score was 10.35, significantly higher than the pre-operative score of 7.58. For the social outcome domain, the post-operative mean score was 3.60, compared to a pre-operative score of 3.15. In contrast, the intimate domain showed a post-operative mean score of 2.65, compared to a pre-operative score of 2.33. The mean FOSQ scores significantly increased in all domains (*p* < 0.001). Further details are provided in Table 2.

In the individual questions in each domain, there was a higher median score in comparison between pre- and post-operative periods among OSA patients. For example, we can see that 75% of all domains in the FOSQ scores for OSA patients had a score between 3 and 4 for the post-operative period in comparison to 2 and 3 scores during the pre-operative period. They had a significant improvement (*p* < 0.001) in all domains (Table 3).

No statistically significant difference between males and females in comparison to the mean percentage change from baseline FOSQ scores between males and females. It found that the mean score in females is higher post-operative in general productivity and vigilance domains in comparison to pre-operative (Table 4).

## Discussion

The present study aimed to assess the impact of BRP on the QOL in patients with OSA, as measured by the FOSQ. The findings demonstrated statistically significant improvements in all domains of the FOSQ, indicating that BRP not only alleviates the physical symptoms of OSA but also enhances patients’ overall well-being and daily functioning. These improvements were evident across multiple domains, including general productivity, activity levels, vigilance, social outcomes, and intimacy. This outcome aligns with the primary goal of BRP, which is to reduce the collapsibility of the upper airway and improve airflow during sleep.

The significance of these findings is underlined by the widespread benefits observed across both functional and social aspects of life. Research shows that OSA not only causes physical fatigue but also leads to considerable psychosocial impairments, including reduced productivity and social interactions.^10^ By demonstrating improvements in these areas, the present study confirms that BRP can effectively mitigate these broader effects of OSA. Moreover, the large improvements in general productivity and vigilance are particularly notable, as they are closely linked to reductions in daytime sleepiness—a major complaint in OSA patients.^12^

The study cohort consisted of 40 participants, predominantly male, with an average age of 44.5 years. The mean BMI of the participants ranged from overweight to obese, which is consistent with the demographic profile of OSA patients described in previous studies.^10,11^ Similar studies have shown that males are more frequently affected by OSA than females, which is reflected in the male-to-female ratio observed in this study.^8^ The correlation analysis revealed an inverse relationship between age, post-operative BMI, and several domains of QOL, such as general productivity and social outcomes, although the correlation coefficients were relatively low. This indicates that while these factors may influence post-operative outcomes, they do not serve as strong predictors of quality-of-life improvement.

The significance of these inverse correlations is relatively limited, as they do not show a strong predictive relationship with post-operative QOL improvements. This finding is consistent with other studies, which found that while demographic factors like age and BMI are important in OSA pathology, their direct influence on post-operative QOL is often secondary to improvements in airway patency and sleep quality.^2,13^ These results suggest that while age and BMI may play a role in recovery, the primary driver of quality-of-life improvement in OSA patients undergoing BRP remains the mechanical improvement in breathing during sleep.

The results of this study are consistent with the literature on quality-of-life improvements following surgical interventions for OSA. A systematic review by Moyer et al.^8^ highlighted the positive impact of surgical interventions, including BRP, on quality-of-life measures, particularly in the domains of general productivity and vigilance.^8^ Furthermore, previous research by Wadhera et al.^10^ demonstrated that patients undergoing BRP experienced significant improvements in both daytime functioning and social interactions, like the findings of the current study.^10^ The statistically significant increase in post-operative FOSQ scores across all domains in the present study supports these conclusions, further emphasizing the effectiveness of BRP in enhancing QOL.

The significance of these findings lies in the breadth of the improvement observed across all domains of the FOSQ, suggesting that BRP has a comprehensive effect on patients’ lives. This is consistent with Pianta et al.,^14^ who emphasized that the primary goal of surgical interventions like BRP is to improve not only physical symptoms but also psychosocial outcomes.^15^ The strong improvements in vigilance and general productivity, both of which are crucial for daytime functioning, underscore BRP’s role in reducing the burden of OSA on daily activities.^10^

In particular, the most substantial improvements were observed in the domains of vigilance and general productivity, with mean differences of 2.78 and 1.45, respectively. These findings are in line with research conducted by Hsiao et al.,^16^ which reported a significant reduction in daytime sleepiness and enhanced cognitive function in patients following BRP.^2^ Similarly, a study by Wadhera et al.^10^ found that patients experienced marked improvements in social outcomes and intimacy, though these domains showed relatively smaller gains compared to vigilance and general productivity.^3,17,18^ The moderate correlation between these domains, as observed in the present study, suggests that improvements in one aspect of QOL may positively influence others.

Interestingly, no statistically significant gender differences were observed in the mean percentage change from baseline FOSQ scores between males and females. This finding is somewhat contrary to prior research,^13^ which indicated that females may experience more pronounced improvements in certain quality-of-life domains post-operatively. However, the relatively small sample size and the male predominance in the present study may have contributed to the lack of observed gender differences.

### Limitations and strengths

One of the primary limitations of this study is the quasi-experimental design, which lacks randomization and a control group. Without random assignment, it is difficult to completely rule out confounding variables, such as natural improvements over time or external influences, which could affect the outcomes. Moreover, the relatively small sample size (*n* = 40), predominantly composed of male participants, limits the generalizability of the findings. Larger, more diverse studies with control groups would provide more robust evidence regarding the efficacy of BRP.^2^ Additionally, the short follow-up period may not capture long-term effects, further restricting the scope of the conclusions that can be drawn from this research.

Despite its limitations, this study has several strengths. It provides important insights into the post-operative QOL in patients with OSA, using a validated and comprehensive measurement tool (FOSQ) that evaluates multiple domains of daily functioning. The study also contributes to a growing body of evidence supporting the benefits of BRP, specifically highlighting its positive effects on both physical and social outcomes. Additionally, the significant improvements observed across all FOSQ domains reinforce the importance of surgical interventions in managing the multifaceted impacts of OSA, including vigilance and social functioning, which are often neglected in clinical evaluations.^11^

## Conclusion

BRP is an effective surgical intervention for improving the QOL in patients with OSAS. This study demonstrated that BRP significantly enhances patients’ health-related QOL, particularly in terms of general productivity, social interactions, mood, daily activities, and overall sleep quality. The improvements observed across these domains are clinically relevant and reflect a meaningful impact on both the physical and psychosocial aspects of life for OSAS patients. Future research should focus on larger, multi-center studies with diverse populations to confirm the generalizability of these findings. Additionally, incorporating long-term follow-up assessments would provide valuable insights into the durability of the observed quality-of-life improvements. Comparative studies evaluating BRP against other surgical and non-surgical interventions could also clarify its relative efficacy and utility within a broader treatment landscape. Exploring the cost-effectiveness of BRP in managing OSAS would further contribute to its clinical and economic evaluation. These findings support the role of BRP as a comprehensive treatment for OSAS, offering significant benefits beyond symptom relief. By addressing these research gaps, future studies can help refine patient selection criteria and optimize clinical outcomes for individuals undergoing BRP.

## Authors’ contribution

FA: writing, data correction, original draft; MK: methodology, review, editing; HM: methodology, review, editing; ARA: review, editing.

## Data availability statement

The data that support the findings in this study are available from the corresponding author upon reasonable request.

## Acknowledgments

The authors would like to thank Maya Thomas and Saritha Shetty, who are the staff nurses dedicated to the snoring and sleep surgery clinic in Al-Khor Hospital, for the distribution of the research questionnaire and data entry, which was a great help. Special thanks to Dr. Prem Chandra: pchandra@hamad.qa, specialist data analyst, for his great effort in providing comprehensive and valuable research and statistical analysis. The authors also wish to acknowledge the medical research center for their ethical approval and support.

## Conflicts of interest

No potential conflicts of interest were reported by the authors.

## Figures and Tables

**Table 1.** Correlation between variables.

**Variables**	**Age**	**BMI^⁑^**	**BMI^⁂^**	**General productivity**	**Activity level**	**Vigilance**	**Social outcomes**	**Intimate**
Age		0.059	0.179	−0.142	−0.123	0.079	−0.142	0.322
BMI^⁑^			0.557^**^	0.247	0.162	0.330*	0.169	0.343*
BMI^⁂^				−0.187	−0.280	−0.005	−0.347*	0.207
General productivity^***^					0.609^**^	0.616^**^	0.541^**^	0.610^**^
Activity level^***^						0.755^**^	0.450^**^	0.500*
Vigilance^***^							0.519^**^	0.698^**^
Social outcomes^***^								0.233
Intimate^***^								

*Correlation is significant at the 0.05 level (2-tailed), **Correlation is significant at the 0.01 level (2-tailed), ***% Change from baseline.

^⁑^(pre-operative), ^⁂^(post-operative), BMI: body mass index.

**Table 2.** Comparison of FOSQ mean scores between pre- and post-operative periods among OSA patients.

**Dimension**	**Pre-operative**	**Post-operative**	**Mean difference (95% CI)**	***p*-value**
General productivity	5.63 ± 1.33	7.08 ± 1.12	1.45 (1.0, 1.9)	<0.0001*
Activity level	8.65 ± 1.69	10.58 ± 1.36	1.92 (1.4, 2.4)	<0.0001*
Vigilance	7.58 ± 2.0	10.35 ± 1.61	2.78 (2.2, 3.4)	<0.0001*
Social outcomes	3.15 ± 0.62	3.6 ± 0.59	0.45 (0.2, 0.7)	<0.0001*
Intimate	2.33 ± 1.27	2.65 ± 1.29	0.33 (0.2, 0.5)	<0.0001*

**Table 3.** Comparison of FOSQ scores between pre- and post-operative periods among OSA patients dimension.

**Dimension**		**Pre-operative**	**Post-operative**	*p*-value*
		Median (IQR)	Median (IQR)	
General productivity	Question 1	2.5 (2, 3)	3.5 (3, 4)	<0.001*
	Question 2	3 (3, 3.75)	4 (3, 4)	<0.001*
Activity level	Question 3	3 (3, 3.75)	4 (3, 4)	<0.001*
	Question 4	2 (2, 3)	3 (3, 4)	<0.001*
	Question 5	3 (3, 4)	4 (3, 4)	0.002*
Vigilance	Question 6	2 (3, 4)	3 (3, 4)	<0.001*
	Question 7	2 (3, 4)	3 (3, 4)	<0.001*
	Question 8	3 (3, 3)	4 (3, 4)	<0.001*
Social outcomes	Question 9	3 (3, 4)	4 (3, 4)	<0.001*
Intimate	Question 10	2.5 (2, 3)	3 (2, 3.75)	<0.001*

IQR: inter-quartile range, *p-value computed using Wilcoxon signed-rank test.

**Table 4.** Comparison of mean percentage change from baseline FOSQ scores between males and females.

**Dimension**	**Gender**	**Change from baseline FOSQ scores**		***p*-value**
		**Mean**	**SD**	
General productivity	Male	31.85	38.23	0.806
	Female	36.90	44.22
Activity level	Male	26.46	24.26	0.514
	Female	18.06	23.73
Vigilance	Male	47.30	49.49	0.737
	Female	38.69	30.14
Social outcomes	Male	18.52	27.83	0.480
	Female	8.33	16.67
Intimate	Male	19.44	29.14	0.195
	Female	0.00	0.00
